# Physicochemical Characterization, *in vitro* Release and Permeation Studies of Respirable Rifampicin-Cyclodextrin Inclusion Complexes

**DOI:** 10.4103/0250-474X.59545

**Published:** 2009

**Authors:** J. S. Patil, Sarasija Suresh

**Affiliations:** Department of Pharmaceutics, Al-Ameen College of Pharmacy, Hosur Road, Bangalore-560 027, India; 1 Department of Pharmaceutics, B. L. D. E. A's College of Pharmacy, BLDE University Campus, Bijapur -586 103, India

**Keywords:** Rifampicin, β-cyclodextrins, inclusion complexes, *in vitro* release, spray drying

## Abstract

The inclusion complexes of rifampicin with sucralose and β-cyclodextrins were prepared by spray drying method. The complexes were characterized by size analyses, scanning electron microscopy, differential scanning calorimetry and x-ray diffraction methods. The results indicated the amorphous nature of resultant products. The solubility, *in vitro* release and skin permeation of the drug were enhanced after formation of inclusion complexes. The *in vitro* release and permeation of the inclusion complexes were greater in simulated lung fluid as compared to pure drug.

Tuberculosis (TB), a ubiquitous, highly contagious chronic granulomatous bacterial infection, is still a leading killer of young adults worldwide. TB has returned with a new face and the global scourge of multi-drug resistant TB (MDR TB) is reaching epidemic proportions[1]. TB is treated with a multi-drug regimen, and is thus exceptionally vulnerable to incidences of side effects, unsatisfactory patient compliance and slow improvement of patients[2].

Chemotherapy of TB is complicated by the need of multidrug regimens that need to be administered over long periods. Poor patient compliance is the single most common reason for chemotherapy failure in TB[2]. To minimize toxicity and improve patient compliance, extensive progressive efforts have been made to develop various implant, microparticulate, and various other carrier-based drug delivery systems to either target the site of *M. tuberculosis* infection or reduce the dosing frequency, which forms an important therapeutic strategy to improve patient outcomes[3,4]. The systems under discussion employ either biodegradable polymers or systems requiring removal after use, and can release the drug either by membrane or matrix-controlled diffusion.

Among all the available anti-tubercular agents, although stable by oral route require frequent administration which produces patient non-compliance and systemic side effects due to their long duration usage[5]. The lung is major portal of entry in the majority of cases of tuberculosis (TB). The problems and issues surrounding the treatment of TB infections and disease are more complex because of body defense appears to have little effect on the organism and poor lung permeation of available drugs[6].

One of the most non-invasive approaches to the drug delivery is via inhalation. Particulate nano-carriers have been praised for their advantageous drug delivery properties in the lung such as avoidance of macrophage clearance mechanism and lung residence time. Unwanted systemic absorption of drugs delivered for the local treatment of respiratory disease is well documented[7,8]. The high frequency of pulmonary tuberculosis demands the development of novel drug delivery approaches that enhances the bioavailability of the drug at the level of lungs.

Complexation with cyclodextrins (βCD) has been reported to enhance the solubility and permeation of poorly water soluble drugs, through the biological membranes. They act as permeation enhancers by carrying the drug through the aqueous barriers which exists before the lipophilic surface of biological membranes[9]. The βCD inclusion complexes containing rifampicin (RFP) may be delivered to lungs by pulmonary route to improve the solubility and permeation of drug, for better treatment of TB. Hence, the present study was planned with an aim of designing, characterization and evaluation of RFP-βCD inclusion complexes for improved therapeutic effect of poorly soluble rifampicin for lung delivery.

## MATERIALS AND METHODS

Rifampicin (RFP) IP was obtained as gift sample from Micro Labs Ltd. Hosur, Tamil Nadu, India. Sucralose (SL) and β-cyclodextrin (βCD) were obtained from Alkem Laboratory, Mumbai, India. Male Wistar rats weighing between 150-200 g were obtained from central animal house, Al-Ameen College of Pharmacy, Bangalore, India. Double distilled water was used throughout the study and all other chemicals were used of analytical grade and used without further purification.

### Phase solubility studies:

The phase solubility study was performed according to the method reported by Higuchi and Conners[10]. An excess amount of RFP was added to the aqueous solution of βCD at various concentrations (5 to 18 mM). The contents were stirred for 72 h at 30°±1. After equilibrium, the samples were filtered and absorbance was read at 475 nm. The apparent stability constant was calculated from the initial straight portion of the phase solubility diagram using the following Eqn., K_1:1_ = Slope/So (1-Slope), where slope is obtained from the graph and So is the equilibrium solubility of RFP in water.

### Formulation of spray dried inclusion complexes:

RFP, βCD, and SL were weighed in the ratios of 2:1:1 (RFP: βCD: SL) (F1) and 2:1 (RFP: βCD) (F2). The RFP was dissolved in 50 ml of isopropyl alcohol with constant stirring and this solution was added to a solution of βCD and SL previously dissolved in 50 ml purified water, this solution was ultrasonicated for 5 min (Probe sonicator, Toshiba, India). The solution was fed to mini spray drier (Lab ultima-222, Mumbai, India) and sprayed into the chamber from a nozzle with diameter 0.7 mm under the atomization pressure of 1.5 kg/cm^2^ with a feed rate of 3 ml/min. The inlet temperature was kept at 150° and outlet temperature at 50±5°. The vacuum in the system was 60 mm Wc and aspirator at 45%. The same procedure was adopted to prepare spray dried form of pure drug also. The product thus obtained was collected, packed and doubly wrapped in aluminum foil and stored in dessicator until further characterization.

### Characterization of spray dried formulations:

Drug content in the spray dried formulations was estimated using UV spectrophotometric method based on the measurement of absorbance at 475 nm in phosphate buffer pH 7.4. Known amount of formulations were dissolved in 50 ml phosphate buffer pH 7.4. Then the amount of drug present in the solution was estimated with suitable dilutions using UV/Vis spectrophotometer (Pharmaspec 1700, Shimadzu, Japan). The spray dried formulations were subjected for particle size analysis using Mastersizer-2000 Ver 2.0 (Malvern Instruments, Malvern U.K.) with a range of detection of 0.02 to 2000 μ. Data analysis was done with Mastersizer 2000 Ver 5.22. Mean particle size was then calculated.

Surface morphology of the particles was investigated using scanning electron microscope (SEM, Jeol, JSM-6360, Japan) with a 10-kv accelerating voltage. The surface of the samples was previously made conductive in a sputtering apparatus. The formulations were sputter coated with platinum and mounted onto the stub of SEM instrument and the particles were focused at different magnifications at room temperature. Thermal behavior of each inclusion complex was examined by using a thermal analyzer (Mettler Toledo, 823^e^, Japan). The examinations were made in the temperature range of 30 to 400°.

X-Ray diffraction (XRD) analysis of pure RFP and spray dried formulations was performed using an X-ray diffractometer (Bruker Axs D8 Advance, Germany). The scanning rate was 4°/min. The voltage/current used was 40 kV/50 mA and the target/filter (monochromator) was copper.

### Aqueous solubility studies:

The aqueous solubility of inclusion complexes were determined at 37±0.5° in pH 7.4 buffer and distilled water. Solubility was measured by soaking a well dispersed powder in excess with solvent until equilibrium was attained. Solute and solvent were placed in the stoppered conical flasks immersed in thermostated water bath shaker (Remi RS-B12 Mumbai, India) and agitated continuously for 24 h at 37±0.5°. After 24 h, solution was filtered through nylon disc filter (0.45 μ) and diluted sufficiently with solvent and absorbance was recorded at 475 nm[11].

### *In vitro* drug release studies:

Drug release from inclusion complexes, pure drug and spray dried pure drug was carried out using modified dissolution method. The media was 0.05 M phosphate buffer solution, 200 μg/ml ascorbic acid added as an antioxidant to prevent oxidative degradation. A known mass of sample was suspended in tubes of buffer solution at two different pH values, 5.2 and 7.4 to examine the effect of pH on drug release. Three replicates were used for each pH values. The tubes were placed in a shaker bath at 37° running at 90 cycles/min. At selected time intervals the samples were withdrawn and filtered through nylon disc filter (0.45 μ) and absorbance was measured at 475 nm[12,13].

### Permeation study:

A permeation study of pure drug and spray dried formulations was carried out across the rat abdominal skin as a physiological model. Abdominal skin of the rat was excised after anaesthetizing and sacrificing. Surface hair of the skin and adhered epidermal tissues were removed carefully without damaging the skin. Then the skin was mounted on the donor compartment of the Keshary-Chien diffusion cell having a downstream volume of 25 ml. The receptor medium used was 0.05 M phosphate buffer solution of pH 5.2. The 200 μg/ml ascorbic acid added as an antioxidant to prevent oxidative degradation. The diffusion was carried out at 37±1.0° and at 50 rpm for 6 h. Five milliliter samples were removed periodically and estimated the drug content spectrophotometrically at 475 nm[14]. The institutional animal ethical committee permission was obtained prior to carry out the experiments (AACP/IAEC/P-34/2006. Dt 26.05.2006).

## RESULTS AND DISCUSSION

**Fig. 1:**
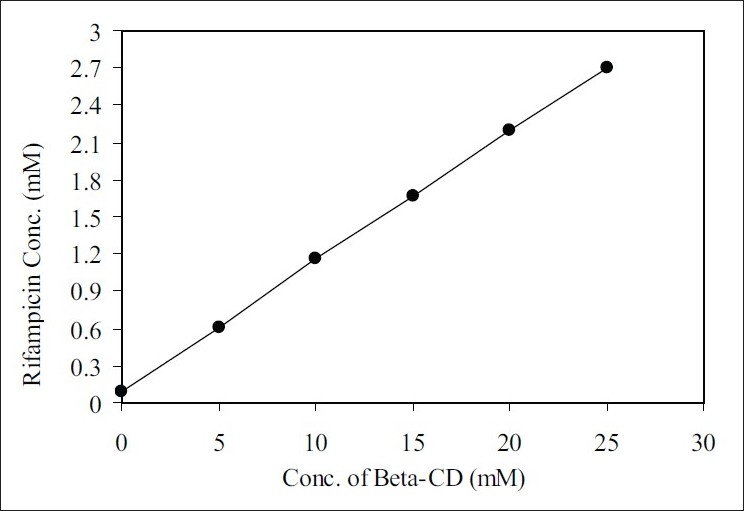
Phase solubility diagram of Rifampicin and βCD. Slope = 0.107, Intercept = 0.045 and R^2^= 0.999.

In the pre-formulation study, the phase solubility was carried out on the RFP with βCD. The results showed that it followed A_L_ type phase solubility as shown in fig. 1. The slope of the line of phase solubility is lesser than the unity which indicates that the stoichiometry is 1:1 for drug: complexing agent. The apparent 1:1 stability constant was calculated and it was found to be 99.27±26.42.

The spray dried formulations obtained were smooth and freely flowing. Spray dried pure drug showed a drug content of 99.7%, whereas inclusion complexes F1 and F2 showed 91.32% and 90.62%, respectively. The mean particle size for spray dried pure drug was 5.59 μ however, for F1 and F2 the particle size was 7.56 μ and 6.58 μ, respectively (Table 1).

**TABLE 1:** DATA OBTAINED FROM THE EVALUATION OF SPRAY DRIED FORMULATIONS

Formulation codes	Ratio	% yield	Drug content (%)	Mean particle size (μ)	Aqueous Solubility (mcg/ml)	% drug permeated per sq.cm of the skin at 6 h.
SPDPD	Spray dried pure drug	60.88	99.70	5.591	1.958	33.35
F1	2:1:1 RFP: SL:BCD	74.50	91.32	7.563	2.657	87.36
F2	2:1 RFP: BCD	78.50	90.67	6.575	2.589	85.87

SPDPD: Spray dried pure drug, F1: Spray dried formulation containing RFP: SL: βCD (2:1:1), F2: Spray dried formulation containing RFP: βCD (2:1)

**Fig. 2:**
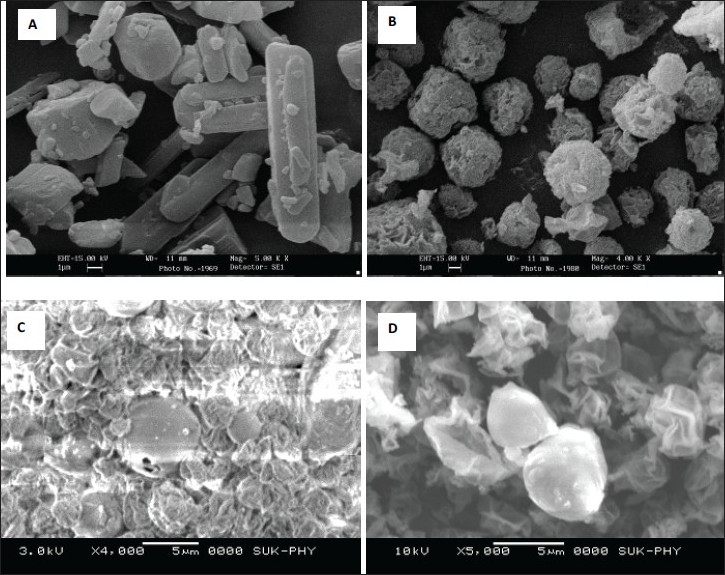
Scanning electron microscopic photographs. Pure RFP (A), spray dried pure RFP (B), formulation F1 (C) and formulation F2 (D).

SEM study revealed that, the pure drug has shown more crystalline nature of its typical and regular size, whereas, spray dried pure drug has shown lesser crystals and there was a formation of spherical drug particles having porous morphology. But the inclusion complexes F1 and F2 have shown small sized particles/aggregates with wrinkles on the surface which suggests the resulting product is amorphous. The aggregation may be due to the presence of βCD and SL (fig. 2).

**Fig. 3:**
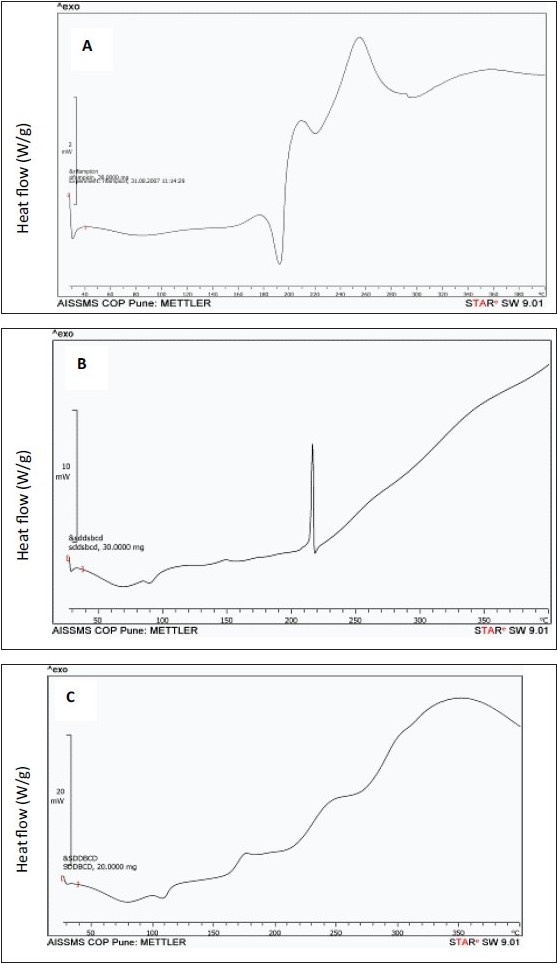
Differential Scanning Calorimetric thermograms. Pure RFP (A), formulation F1 (B) and formulation F2 (C).

DSC thermograms of pure drug, formulation F1 and F2 are shown in fig. 3. These results indicated that the pure drug has shown an endothermic peak at 190°, which is due to melting of the drug. The thermograms of F1 has shown a broad peak at 90°, which may be due to loss of associated water, whereas, another sharp exothermic peak at 215° may be due to the evolution heat as a result of decomposition of sucralose. While, in the thermograms of F2, this exothermic peak at 215° is not observed because of the absence of sucralose and a broad peak at 110° is because of loss of associated water in βCD. However, the sharp endothermic peak of drug has not appeared in both F1 and F2 indicating that the drug is in amorphous and complex form with the βCD.

**Fig. 4:**
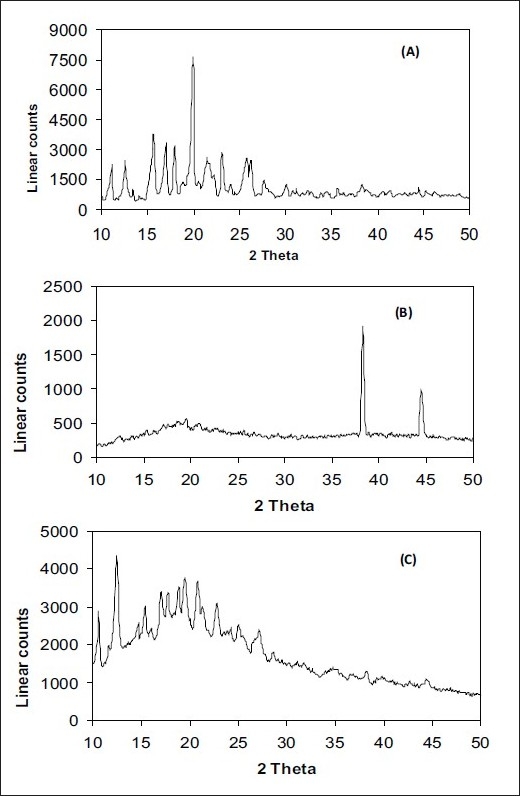
X-ray diffractograms. Pure RFP (A), formulation F1 (B) and formulation F2 (C).

X-ray diffractograms of pure drug has shown its own characteristic crystal peaks between 2θ of 10° and 25° (fig. 4). Whereas, these characteristic peaks of drug were absent in spray dried formulations F1 and F2, indicating the amorphous nature and complex formation between drug and complexing agents. Inclusion complexes showed undefined, broad, diffused peaks with the low intensities. This indicated that the inclusion complexes obtained were not of crystalline nature and almost complete amorphization of drug has occurred.

**Fig. 5:**
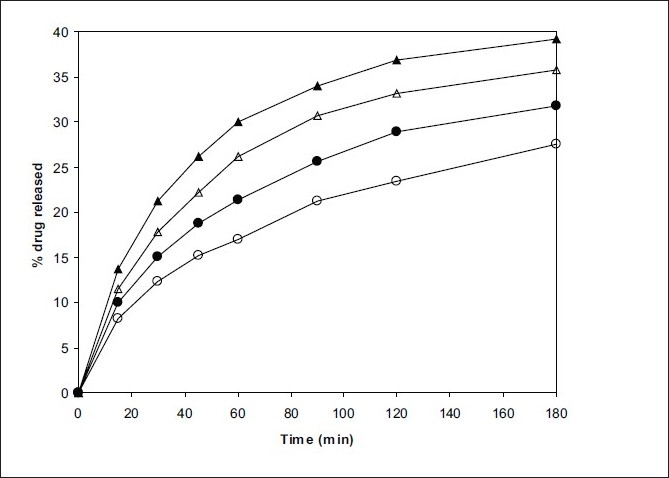
*In vitro* drug release profile in pH 5.2 and pH 7.4 media. Pure RFP (–●–,–○–) and spray dried pure RFP (–▲–,–△–). Closed symbols indicate release in pH 7.4 and open symbols indicate the release in pH 5.2 media.

**Fig. 6:**
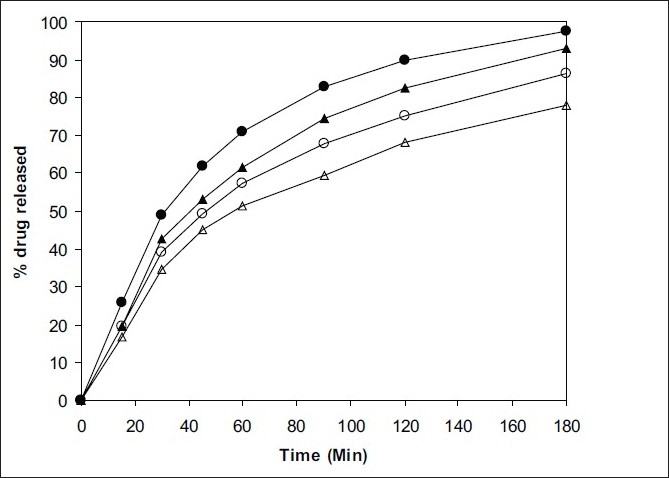
*In vitro* drug release from spray dried formulations in pH 5.2 and pH 7.4 media. F1 (–●–,–○–) and F2 (–▲–,–△–). Closed symbols indicate release in pH 7.4 and open symbols indicate the release in pH 5.2 media.

Solubility was enhanced after the spray drying process and complex formation. The F1 formulation has shown maximum solubility than the F2 (Table 1). Dissolution profiles of pure drug, spray dried pure drug, F1 and F2 formulation are shown in figs. 5 and 6. The drug release study was carried out in both simulated physiological pH 7.4 and endosomal pH of alveolar macrophages (5.2). Dissolution in simulated lung fluid of pH 5.2 (SLF) without enzyme was studied to gain information about the dissolution of drug in the pulmonary epithelium. The dissolution of drug from the spray dried complexes was improved as compared to pure drug. However, the dissolution was greater in physiological media as compared to lung's media in all the cases. The pure drug in its native form has shown 27.5% release, whereas, in complex form it has shown enhanced dissolution; there was almost 4 fold increase in dissolution rate in pH 5.2 media. The improvement in dissolution rate of inclusion complexes may be attributed to the degree of amorphization of the drug together with increase in both the wettability and the solubility of the drug.

The permeation study of the complexes in SLF without enzyme was studied. The rat skin was choosen as a physiological model just to know the permeation characteristics of drug alone and from prepared inclusion complexes. The skin permeation study of spray dried pure drug and complexes F1 and F2 was carried out and results are given in Table 1. The permeability across rat skin was improved after complexation and the permeation was enhanced by almost three folds. A spray dried inclusion complexes of RFP with SL and βCD were prepared in the ratios of 2:1:1. The inclusion complexes were characterized by size analyses, SEM, DSC and XRD. The results indicated the amorphous nature of resultant products. The solubility, *in vitro* release and skin permeation of the drug were enhanced after formation of inclusion complexes. The *in vitro* release and permeation of the inclusion complexes were greater in SLF as compared to pure drug.
